# Distribution of HCV Genotypes Among People Who Inject Drugs in Tunisia: New Evidence for Scaling Up Prevention and Treatment Toward National Elimination Goal

**DOI:** 10.3389/fmicb.2021.697859

**Published:** 2021-07-27

**Authors:** Anissa Chouikha, Amine Ghrabi, Amira Ghodbane, Walid Hammemi, Marwa Khedhiri, Amel Sadraoui, Henda Touzi, Hichem Ben Hassine, Sonia Maatoug, Chaima Bensaoud, Sonia Abdelhak, Samir Bouarrouj, Mariem Gdoura, Hedia Chaouachi, Henda Triki

**Affiliations:** ^1^Laboratory of Clinical Virology, WHO Reference Laboratory for Poliomyelitis and Measles in the Eastern Mediterranean Region, Pasteur Institute of Tunis, Tunis El Manar University, Tunis, Tunisia; ^2^Association Tunisienne d’Information et d’Orientation sur le SIDA et la Toxicomanie (Tunisian Association for Information and Orientation on HIV/AIDS and Toxicomania – ATIOST), Tunis, Tunisia; ^3^Science Shop, Institut Pasteur de Tunis, Tunis, Tunisia; ^4^Institute of Parasitology, Biology Centre, Czech Academy of Sciences, Ceske Budejovice, Czechia

**Keywords:** hepatitis C virus, injecting drug users, PWID, prevention, treatment, substance abuse, Tunisia

## Abstract

Little is known about the distribution of hepatitis C virus (HCV) genotypes among people who inject drugs (PWID) in North African countries, including Tunisia. This study aims to describe HCV genotypes circulating among Tunisian PWID. A cross-sectional study was conducted, and 128 HCV-positive PWID were recruited between 2018 and 2019 from community-based harm reduction centers. After informed consent, sociodemographic characteristics and risk behavior data were obtained using an interviewer-administrated questionnaire. Blood samples were collected for further serological and molecular testing. Overall, five women and 123 men were included. The median age was 39.5 years. The majority of PWID (56.3%) had less than a secondary level of education, were single (57%), were unemployed (65.6%), were incarcerated at least once (93.0%), and had a history of residency in at least one foreign country (50.8%). During the previous 12 months, 82.0% reported having reused syringes at least once, 43.8% shared syringes at least once, while 56.2% had at least one unprotected sexual relation, and 28.1% had more than two different sexual partners. Tattooing was reported among 60.2%. All positive results for HCV-infection by rapid testing were confirmed by enzyme-linked immunosorbent assay (ELISA). HCV-RNA was detectable in 79.7%. Genotyping showed a predominance of genotype 1 (52%) followed by genotype 3 (34%) and genotype 4 (10%). Four patients (4%) had an intergenotype mixed infection. Subtyping showed the presence of six different HCV subtypes as follows: 1a (53.2%), 1b (6.4%), 3a (33.0%), 4a (3.2%), and 4d (4.3%). This is the first study describing circulating HCV genotypes among PWID in Tunisia. The distribution of HCV genotypes is distinct from the general population with a predominance of subtypes 1a and 3a. These findings can be used to guide national efforts aiming to optimize the access of PWID to relevant HCV prevention and treatment measures including pangenotypic regimens for patients infected with HCV genotype 3.

## Introduction

Infection with hepatitis C virus (HCV) remains a major global health problem with significant morbidity and mortality due to cirrhosis and hepatocellular carcinoma. The World Health Organization (WHO) estimates that 71.1 million people are living with HCV worldwide, out of which the majority remain untreated (World Health Organization, 2018b). In 2015, 23% of newly diagnosed HCV infections were attributed to injecting drug use (IDU) (World Health Organization, 2017). Due to sharing of injection equipment, people who inject drugs (PWID) are disproportionately affected by blood-borne viruses including HCV. About 52.3% of the global PWID population (15.6 million) had been exposed to HCV (Degenhardt et al., 2017) and PWID populations accounted for 8.5% of all HCV infections globally (Grebely et al., 2019).

The Global Health Sector Strategy on Viral Hepatitis 2016–2021 of the WHO recognizes harm reduction services for PWID and HCV treatment as key interventions for the elimination of HCV as a global public health threat by 2030 (World Health Organization, 2016). Harm reduction services includes testing for human immunodeficiency virus (HIV), HCV, and hepatitis B virus (HBV) infections (Martin et al., 2013), as well as syringe services programs (SSPs), which provide PWID with sterile syringes to minimize unsafe injecting risk behaviors (Palmateer et al., 2010; MacArthur et al., 2014). The recent advent of highly efficacious direct-acting antiviral (DAA) therapies (Ara and Paul, 2015) and their associated cure and prevention benefits (Martin et al., 2013) provide an unprecedented opportunity to reduce HCV-related morbidity and mortality among PWID (Grebely et al., 2017a).

The Middle East and North Africa (MENA) region has been identified as the most affected region by HCV infection worldwide (World Health Organization, 2017). There are significant variations for national HCV prevalence levels between MENA countries (Chaabna et al., 2018). While 15% of the adults were infected by HCV in Egypt, the overall HCV prevalence across the Maghreb subregion was about 1% with IDU identified as a significant contributor to HCV transmission in these countries (Fadlalla et al., 2015). Notably, in Tunisia, high levels of HCV prevalence among local groups of PWID were estimated, ranging from 21.7 to 29.1% (Fadlalla et al., 2015; Chaabna et al., 2018; Grebely et al., 2019).

In addition, it has been established that HCV genotypes’ distribution varies significantly between PWID and the general population, with IDU as a key vector for the diversification of circulating viral genotypes (Ruta and Cernescu, 2015). A systematic review published in 2016 concluded that HCV genotypes 1a and 3 were the most common among populations of PWID globally (Robaeys et al., 2016). While the available estimations for the MENA region were limited to five countries only, this trend was confirmed in neighboring Libya and Morocco where genotypes 1 and 3 accounted for 86.6 and 90.5% of PWID, respectively (Alashek and Altagdi, 2008; Trimbitas et al., 2014). In Tunisia, genotype 1b had been identified as the most common genotype in the general population (79.5–82.6%) followed by genotype 2 (10.1–13.3%) (Djebbi et al., 2004; Mejri et al., 2005; Gower et al., 2014; Chouikha et al., 2021). However, to our knowledge, there are no published studies describing the distribution of HCV genotypes among PWID up to now. The absence of such studies suggests that the distribution of HCV genotypes in Tunisia might be underestimated especially among PWID groups. Therefore, the aim of this work is to describe the HCV genotypes circulating among a Tunisian population of PWID attending a harm reduction program managed by the Tunisian Association for Information and Orientation on HIV/AIDS and Toxicomania (ATIOST).

## Materials and Methods

### Study Population and Design

Participants were recruited through word of mouth and self-referral at a harm reduction program managed by the community-based organization ATIOST. This program provides peer educators and active PWID with free and confidential access to sterile injection equipment as well as HIV/HCV screening across four harm reduction centers covering the North Eastern regions of Greater Tunis and Bizerta. All participants who (1) provided signed informed consent, (2) had an available positive rapid test for HCV antibody detection, (3) completed an interviewer-based questionnaire, and (4) had an available blood sample collected at one of ATIOST harm reduction centers between February 19, 2018 and January 24, 2019 were considered eligible for inclusion. Sociodemographic characteristics and risk behaviors data were collected at enrollment using an interviewer-administrated questionnaire. Further serologic and molecular testing were performed at the Laboratory of Clinical Virology of IPT.

### Screening for HCV, HIV, and HBV Infection

Hepatitis C virus and HIV antibody detection was performed at enrollment using the appropriate available rapid test (SD Bioline HCV, Labman^®^ HCV Test, or Vikia^®^ Anti-HCV, and Turklab^®^ Anti-HIV 1/2 Test or Alere Determine^®^ HIV–1/2 Ag/Ab Combo). Confirmation of HCV positivity was carried out at the laboratory by commercial third-generation enzyme-linked immunosorbent assay (ELISA) from Murex-Diagnostics-France (Murex anti-HCV) according to the manufacturers’ instructions. Serologic detection of HBV antigen (AgHBs) and HBV antibodies (anti-HBc) was also carried out for confirmed HCV-positive samples using commercial ELISA from BIORAD-Diagnostics according to the manufacturers’ instructions.

### Hepatitis C Virus RNA Quantification and Genotyping

The detection and quantification of HCV RNA was conducted on plasma samples using real-time RT-PCR test from Roche Diagnostics (COBAS^®^ AmpliPrep/COBAS^®^ TaqMan^®^) with a limit of detection of 15 IU/ml [1 International Unit (IU) = 5.81 genome copies). For samples with detectable HCV RNA, genotyping kits of HCV real-time RT-PCR Cobas 4800 HCV were used based on a set of primers that targets core, NS5b, and 5′UTR regions of the HCV genome for genotype identification. It allows the detection of genotypes 1a, 1b, 2, 3, 4, 5, and 6 as well as intergenotype mixed infections. In a next step, a selected subset of samples including non-determined genotypes and/or subtypes was selected to be assessed by nucleotide sequencing in the NS5b gene as described previously (Chouikha et al., 2021). Obtained sequences were submitted to GenBank database under accession numbers MW221786–MW221821 and MW678731–MW678756. Sequence alignment was performed by Clustal W within the software “Molecular Evolutionary Genetic Analyses” (MEGA) software version 7.0.26 and phylogenetic tree was also constructed using by the maximum likelihood method and the kimura-2 parameter model using MEGA software (Kumar et al., 2016). Topology was supported by 1,000 bootstrap replicates.

## Results

**FIGURE 1:**
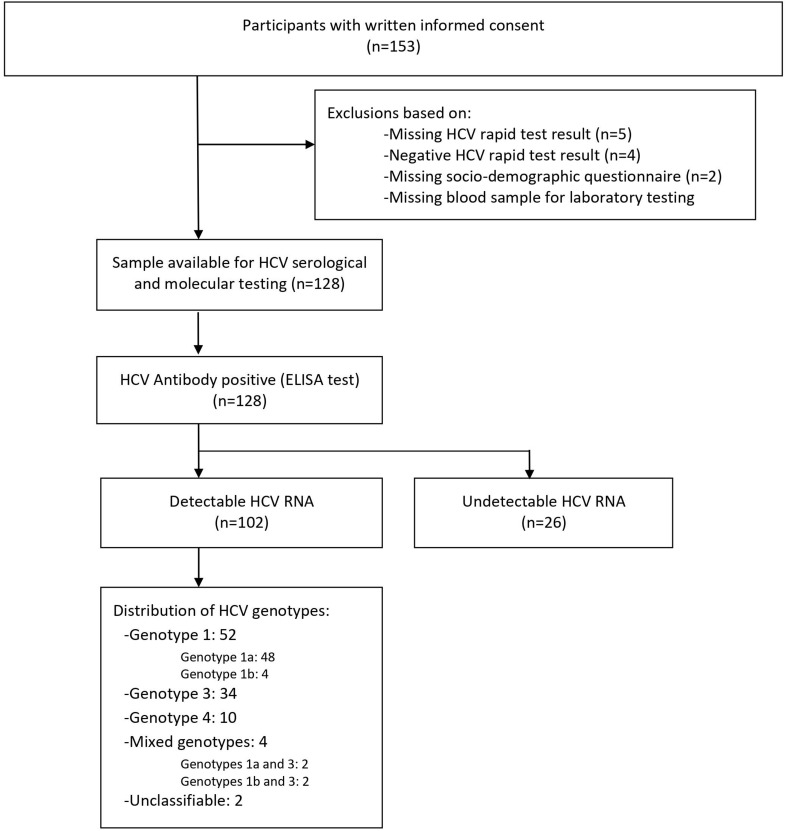
Study flowchart. Overall, 128 participants provided written informed consent. A total of 128 participants had a positive rapid hepatitis C virus (HCV) test and an available sample for serological and molecular testing. All samples with HCV-positive rapid test (100% *n* = 128) were confirmed positive by HCV enzyme-linked immunosorbent assay (ELISA) test. Among confirmed HCV-positive participants, 79.7% (*n* = 102) had a detectable viral RNA. HCV genotypes were identified for 100 people who inject drugs (PWID).

In total, 153 participants signed the informed consent form, out of which 128 had a positive rapid anti-HCV test result at enrollment, completed the interviewer-based questionnaire, and had an available blood sample for further laboratory serological and molecular testing (Figure 1).

### Sociodemographic Characteristics and Risk Behaviors

Sociodemographic characteristics of the study population are described in Table 1. Among the 128 included PWID, there was a total of five women and 123 men. The age ranged from 23 to 61 years with a median value of 39.5 years (25–75 IQR = 34–46). The majority of PWID had less than a secondary level of education (*n* = 72; 56.3%), were single (*n* = 73; 57%), were unemployed (*n* = 84; 65.6%), and were incarcerated at least once (*n* = 119; 93.0%). Moreover, 85.9% (*n* = 110) were living with their family members, while 14.1% (*n* = 18) were living alone. Among 65 participants (50.8%) who had a history of residency in at least one foreign country, specific information regarding countries of residency was obtained for 57 participants. Most of these PWID lived in European countries (*n* = 54), especially Italy (*n* = 47) and/or France (*n* = 27). Only seven PWID had a history of residency at least in one country from the Maghreb region, while five PWID lived in at least one country from the broader MENA region. A history of tattooing was reported among 60.2% (*n* = 77). Regarding injection risk behaviors during the last 12 months, 82.0% (*n* = 105) reported having reused syringes at least once, and 43.8% (*n* = 56) reported having shared syringes at least once. For sexual risk behaviors during the last 12 months, 56.2% (*n* = 72) reported having at least one unprotected sexual relation and 28.1% (*n* = 36) had more than two different sexual partners. Overall, 10.2% (*n* = 13) of HCV-positive PWID had a coinfection with HIV, whereas 3.9% (*n* = 5) were positive for HBsAg, and 83.6% (*n* = 107) were positive for anti-HBc. Based on these results, a previous exposure to all of HIV, HBV, and HCV infections was observed among 9.4% (*n* = 12).

**TABLE 1:** Sociodemographic characteristics and risk behaviors of hepatitis C virus (HCV)-positive people who inject drugs (PWID) attending harm reduction centers in the North Eastern region of Tunisia.

		**Number (*n*)**	**Percentage (%)**
Gender	Male	123	96.1
	Female	5	3.9
Age group in years	20–29	8	6.3
	30–39	56	43.8
	40–49	40	31.3
	50–59	22	17.2
	> 60	2	1.6
Education level	Primary	72	56.3
	High school	52	40.6
	University	4	3.1
Civil status	Single	73	57
	Married	33	25.8
	Divorced/widowed	22	17.2
Employment	Employed	44	34.4
	Unemployed	84	65.6
History of incarceration	yes	119	93.0
	no	9	7.0
Housing	Living alone	18	14.1
	Living with family members	110	85.9
Residency in a foreign country	Yes	65	50.8
	No	63	49.2
	Yes	77	60.2
History of tattooing	No	51	39.8
Syringe reuse in the last 12 months	Yes	105	82.0
	No	23	18.0
Syringe sharing in the last 12 months	Yes	56	43.8
	No	72	56.2
Unprotected sexual relations	yes	72	56.2
	No	56	43.8
More than one sexual partner	Yes	36	28.1
	No	92	71.9

### Hepatitis C Virus Serological and Molecular Analysis

**FIGURE 2:**
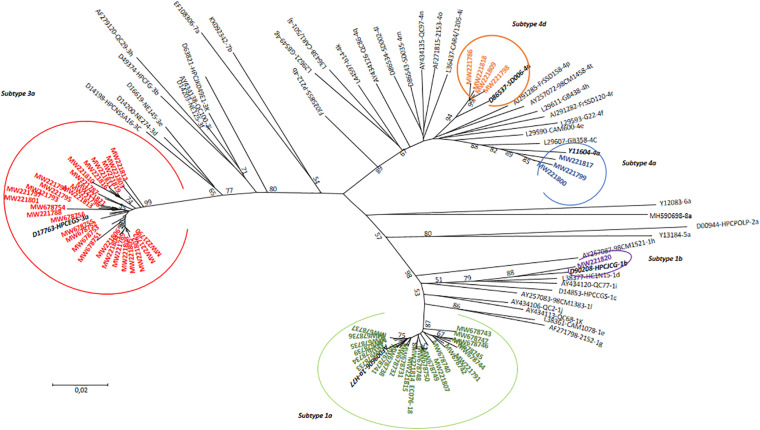
Phylogenetic tree of HCV genotypes identified among HCV-positive PWID attending an SSP in North Eastern region of Tunisia in comparison with 45 reference strains. The tree was performed using the maximum likelihood method and the kimura-2 parameter model. Topology was supported by 1,000 bootstrap replicates. Bootstrap values lower than 50 were not indicated. The sequences reported in this study were identified by the accession numbers.

All positive results for HCV antibody detection obtained by rapid testing (*n* = 128; 100%) were confirmed at the laboratory using ELISA test. Viral HCV RNA was detectable in 79.7% (*n* = 102), with viral load ranging from 25 to 80,200,000 UI/mL. In the first step, genotyping by real-time RT PCR was performed for all 102 samples with detectable HCV RNA. This method allowed the genotype identification for 97 samples, while five samples only could not be genotyped. Samples with identified genotypes included genotype 1a (*n* = 41), 1b (*n* = 4), 1 (*n* = 5), 3 (*n* = 33), and 4 (*n* = 10), in addition to four samples with intergenotype mixed infections with genotypes 1a and 3 (*n* = 2) or 1b and 3 (*n* = 2). In the next step, a subset of 64 (*n* = 64/102; 62.7%) samples was selected for sequencing in the NS5b region. This selection included 46 samples with non-identified genotypes and/or subtypes as well as 18 samples with identified genotype and subtype. Among this selection, 62 HCV sequences were obtained for genotype and subtype identification or confirmation. A phylogenetic tree comparing the obtained HCV sequences with reference strains is shown in Figure 2.

**FIGURE 3:**
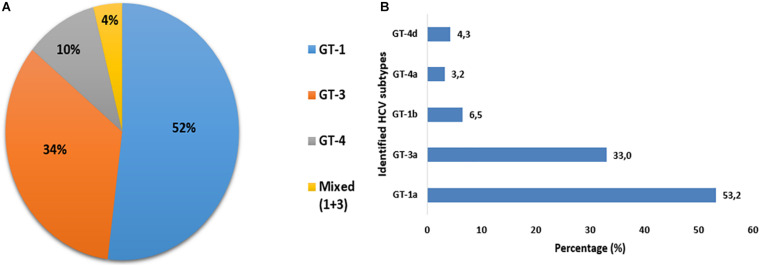
Distribution of HCV genotypes **(A)** and subtypes **(B)** among PWID attending syringe services programs (SSPs) in the North Eastern region of Tunisia.

Out of a total of 102 samples with detectable HCV RNA, 100 (98%) were successfully genotyped, whereas two (2%) samples could not be genotyped. Among HCV-infected PWID with identified genotypes, the predominant genotype was 1 (52%) followed by genotype 3 (34%) and genotype 4 (10%). Four patients (4%) had an intergenotype mixed infection with genotypes 1 and 3. Overall, six different HCV subtypes had been identified among 94 PWID. The HCV subtype distribution was as follows: subtypes 1a was identified in 50 (53.2%), 1b in 6 (6.4%), 3a in 31 (33.0%), 4a in 3 (3.2%), and 4d in 4 (4.3%) (Figure 3).

## Discussion

The present study reports the sociodemographic features, risk behaviors, and the distribution of HCV genotypes in a population of PWID recruited between 2018 and 2019 at a community-based SSP program in the North Eastern region of Tunisia.

One important point to note is that there was a low proportion of women included in the study. This might be explained partly by the fact that women consuming drugs are less likely to openly report their IDU behavior because of significant social and cultural stigma faced by female PWID across North African countries. The difficulty of recruiting female PWID participants was previously reported in Tunisia (Mosbah et al., 2018), Libya (Mirzoyan et al., 2013), and Morocco (Trimbitas et al., 2014). Additionally, this study shows that HCV-infected PWID attending an SSP program were somewhat younger than larger groups of HCV-infected patients from the general Tunisian population (Bettaieb et al., 2019; Chouikha et al., 2021). The age of the studied PWID was close the age of previously studied populations of injection and non-injection drug users (Sellami et al., 2016; Mosbah et al., 2018).

Furthermore, significant social and economic vulnerability was observed in this study. The majority of PWID had a maximum of primary schooling, were unemployed, and had been previously incarcerated at least once. Low education levels and high unemployment rates were previously reported in PWID groups from other countries (Omland et al., 2013; Edmunds et al., 2019). This social and economic vulnerability may enhance exposure to high-risk environments where harmful behaviors can occur. In fact, high prevalence levels of HCV infection have been reported in prison settings where various risky behaviors, such as use of non-sterile injecting equipment, unsafe tattooing, and unprotected sex, remain important drivers of HCV transmission (Larney et al., 2013; Heijnen et al., 2016). In this light, the access of PWID to adequate prevention and treatment services should be strengthened in prison settings to reduce harmful behaviors and to improve the overall health status of PWID groups (Kamarulzaman et al., 2016; Rich et al., 2016).

In the present work, sharing of injection equipment and syringe reuse during the past 12 months was also frequently reported among our PWID. In reference to the international literature, the main factors associated with transmission and acquisition of HCV infection include sharing of injection equipment, early initiation of IDU, and frequent injecting (Zhou et al., 2019). While this study reported significant levels of risky injection behaviors, little is known about the coverage of existing harm reduction interventions in Tunisia. Although SSPs are provided in Tunisia since 2009 (World Health Organization, 2012), these programs have been implemented on a small scale, in a limited number of community-based centers. Thus, data on intervention coverage is not readily available. There is also a low coverage of interventions for HIV and HCV prevention in the MENA region, with less than four syringes are distributed annually per PWID through harm reduction programs (Larney et al., 2017). Furthermore, access to opioid substitution therapies (OSTs) is not provided in many MENA countries including Tunisia. While the WHO and UNAIDS identified SSP and OST as high-priority interventions for PWID, greater investments in evidence-based interventions for HCV prevention are urgently needed in Tunisia.

In addition to HCV, it is estimated that PWID are highly exposed to other blood-borne viruses such as HIV and HBV. A global systematic review estimated that 13% of PWID are living with HCV–HIV coinfection and that 3% are HCV–HBV coinfected (Rashti et al., 2020). Results from the present study showed similar trends regarding HCV–HIV and HCV–HBV coinfections. Thus, HIV and HBV prevention and treatment among PWID should be improved through the development of specific recommendations that are specific to HCV-infected PWID. This includes enhancing systematic screening for HBV and HIV coinfections and increasing HBV vaccination rates in non-immunized PWID groups.

From the virological side, patients included in this study were recruited based on HCV testing using rapid tests at the SSP facility level. Positive results for HCV rapid test were confirmed by ELISA testing for all participants. This is in concordance with previous findings showing a high performance of rapid tests in screening of HCV infection especially in high-risk persons (Shivkumar et al., 2012; Poiteau et al., 2016; Barbosa et al., 2017; Mane et al., 2019; Sharafi et al., 2019). Rapid tests represent an interesting alternative for HCV screening among high-risk populations given the advantage of simplicity, quick performance at room temperature, and the limited need of equipment and training for health professionals. Furthermore, point-of-care HCV RNA viral load tests are becoming increasingly available, allowing quick and reliable detection of active HCV infection from venipuncture and finger-stick capillary whole-blood samples without requiring special laboratory infrastructure (Wang et al., 2011; Cooper, 2017; Grebely et al., 2017b; Saludes et al., 2020). In particular, these point-of-care diagnostics for HCV antibodies and HCV RNA are interesting quick and easy testing tools that should be used as part of the national elimination strategy in Tunisia to expand access to HCV diagnosis and treatment among hard-to-reach groups such as PWID. In the present study, 80% had a detectable HCV-RNA, reflecting a high rate of active viral replication within Tunisian PWID. While little is known about rates of chronic HCV infection in Tunisia, especially among high-risk groups, a recent study showed a detectable viral RNA rate of 56.8% in a Center – western city (Thala) in Tunisia (Bettaieb et al., 2019). Higher rates (79%) were also observed among groups of Tunisian hemophiliacs (Djebbi et al., 2008). Furthermore, a systematic review conducted in 2019 highlighted the lack of data for the MENA region and the need to improve country-level estimates for viremic HCV prevalence among PWID (Grebely et al., 2019). Generally, high rates of chronic HCV infection are associated with PWID populations because of recurrent exposure to HCV infection (Midgard et al., 2016). HCV chronicity levels among PWID in 12 European countries ranged between 53 and 97% (Wiessing et al., 2014).

Hepatitis C virus genotype distribution among PWID is completely different from that observed in the Tunisian general population. HCV genotypes 1 and 3 were the most prevalent genotypes (85%) with a predominance of subtype 1a overall. In contrast, previous studies conducted in the general population, either in Tunisia or in some neighboring countries from the Maghreb region, have shown a predominance of subtype 1b followed by subtype 2c (Djebbi et al., 2003, 2004; Mejri et al., 2005; Ezzikouri et al., 2013; Bettaieb et al., 2019; Chouikha et al., 2021). In this study, there were very few cases of subtype 1b detected among PWID, while genotype 2 was not detected at all. High prevalence of genotypes 1a and 3a among PWID was reported globally (Ruta and Cernescu, 2015; Robaeys et al., 2016; Grassi and Ballardini, 2017). This difference in genotype distribution could be explained by the fact that the majority of participants had a history of residency in a foreign country, mainly in Italy and France, where subtypes 1a and 3a were widely detected in PWID groups (Bourlière et al., 2002; Sereno et al., 2009; Trimbitas et al., 2014; Bielen et al., 2017). Thus, it is likely that PWID, with their history of residency in foreign countries, contribute to the introduction of additional HCV subtypes in Tunisia.

We also found in our series that four participants (4%) had an intergenotype mixed infections with genotypes 1 and 3. In fact, PWID are also likely to be exposed to multiple HCV infections due to recurrent risky injection behaviors. The prevalence of intergenotype mixed infections ranged from 14 to 39% (Cunningham et al., 2015).

In addition, sequencing-based methods are increasingly used to identify subtypes when RT-PCR or hybridization tests give non-conclusive results, to identify already new genotypes/subtypes that may emerge and to differentiate between genetically different variants within HCV subtypes. In the present work, sequencing and phylogenetic analysis allowed us to genotype three of the five not typed ones by qRT-PCR, and to determine subtypes within genotypes 1, 3, and 4 by comparison with published reference sequences. The association of multiple techniques for HCV genotyping especially for the high-risk populations could be of great interest to better assess the right genotype distribution and to guide therapeutic approach.

It is noteworthy that national guidelines for the introduction of the DAA regimens in Tunisia were developed in 2015 based on sofosbuvir/ledipasvir as a preferred first-line regimen (Société Tunisienne de Pathologie Infectieuse and Société Tunisienne de Gastro-entérologie., 2015). This is a non-pangenotypic regimen, which is commonly used in countries where only a single HCV genotype like genotype 1 is widely isolated (Zoratti et al., 2020). In the current DAA era, HCV genotype 3 infection has emerged as relatively difficult to treat when compared with other HCV genotypes, causing lower sustained virologic response rates and longer treatment duration (Ampuero et al., 2014). Patients with HCV genotype 3 infection had an accelerated fibrosis progression and higher incidence of hepatocellular carcinoma (Bochud et al., 2009; Nkontchou et al., 2011; Kanwal et al., 2014; McMahon et al., 2017). As HCV genotyping remains a key component of pretherapeutic assessment prior to treatment initiation, national guidelines recommend treating HCV genotype 3 infection with sofosbuvir–ribavirin or with sofosbuvir/daclatasvir for 24 weeks among patients without cirrhosis and with sofosbuvir/daclatasvir–ribavirin for 24 weeks among patients with compensated or decompensated cirrhosis even though daclatasvir is not available in Tunisia. The WHO guidelines for HCV treatment were updated twice since 2016 to include pangenotypic DAA regimens reducing the need for genotyping to guide treatment decisions and recommending sofosbuvir/daclatasvir combination for genotype 3 infection during 12 weeks for patients without cirrhosis and 24 weeks for patients with compensated cirrhosis (World Health Organization, 2018a). Current European recommendations advice for the use of pangenotypic regimens of sofosbuvir/velpatasvir for 12 weeks or glecaprevir/pibrentasvir for 8 weeks for patients infected with HCV genotype 3 and without cirrhosis, and with glecaprevir/pibrentasvir for 12 weeks for patients with compensated cirrhosis (European Association for the Study of the Liver, 2018). Due to the potential suboptimal treatment outcomes associated with currently available DAA regimens, in addition to the high rate of genotype 3 reported in the present study, the introduction of pangenotypic treatments, such as sofosbuvir–daclatasvir, sofosbuvir/velpatasvir, and glecaprevir/pibrentasvir, would be highly beneficial to maximize access to more efficacious and shorter HCV treatments in Tunisia especially for PWID with HCV genotype 3 infection.

While this study illuminates significant knowledge gaps regarding HCV genotype distribution among PWID, these findings cannot be generalized to the overall population of HCV-infected PWID in Tunisia for several reasons. First, the study was limited to the North Eastern geographic region of the country, and some subgroups of PWID such as youth and women were not adequately represented. In the future, this limitation could be addressed by initiating research partnerships with community-based organizations that provide gender-sensitive harm reduction services, which can be also respondent to adolescents and young people’s specific needs with reduced stigma and discrimination toward drug users. In addition, the study is potentially subject to selection bias because included participants were exclusively recruited within a local SSP where participants were more likely to have benefited from diverse prevention and treatment interventions. On the other hand, interview responses might be affected by information bias since IDU is severely stigmatized and criminalized in Tunisia. To reduce stigma and discrimination toward PWID and minimize information bias, all interviews were administrated by trained medical doctors in close cooperation with peer educators and community representatives. In light of these different strengths and limitations, this study provides the first exploration about HCV genotype diversity among PWID in Tunisia. In conclusion, findings from this study provided the first exploration about the distribution of HCV genotypes among a population of Tunisian PWID, which can be used to improve national efforts aiming to achieve national HCV elimination goal. Given the predominance of HCV subtypes 1a and 3a among PWID, this distribution of HCV genotypes was distinct from the general population. While further genotype surveillance and monitoring studies among vulnerable populations are highly needed to improve the current knowledge of HCV epidemiology in Tunisia, community-based prevention and harm reduction efforts for PWID should be strengthened and expanded, and current national guidelines for HCV treatment should be updated to include the use of new pangenotypic DAA regimens and to ensure access to better treatment outcomes especially for patients infected with HCV genotype 3.

## Data Availability Statement

The datasets presented in this study may be found in online repositories. The names of the repositories and accession numbers can be found in the article.

## Ethics Statement

This study was conducted in accordance with the Declaration of Helsinki and the study protocol was reviewed and approved by the Biomedical Ethics Committee of the Institute Pasteur de Tunis (reference number: 2018/04/I/LVCIPT/V2). All participants provided their written informed consent before enrolment in the study.

## Author Contributions

AC and AGhr contributed to the conception and design of the study, and wrote the first draft of the manuscript. AGho, WH, MK, AS, and HTo contributed in the investigation. HH, SM, CB, and SA contributed in the data curation and resources. SB and MG contributed in the data curation and investigation. HTr contributed in the reviewing, editing the manuscript, and supervising the work. All authors contributed to the manuscript revision, and read and approved the submitted version.

## Conflict of Interest

The authors declare that Prochidia Laboratory provided genotyping kits for free to the Laboratory of Clinical Virology to conduct the present work.

## Publisher’s Note

All claims expressed in this article are solely those of the authors and do not necessarily represent those of their affiliated organizations, or those of the publisher, the editors and the reviewers. Any product that may be evaluated in this article, or claim that may be made by its manufacturer, is not guaranteed or endorsed by the publisher.
